# A Young Mother

**Published:** 1877-04

**Authors:** 


A Young Mother.—An esteemed and entirely trustworthy correspondent has furnished us with the following facts touching a case which came under his observation. As an instance of early maternity, the case is one which certainly vies with any case on record: The girl first menstruated when ten years and six months of age. She became pregnant at eleven years and six months, and was safely delivered of a male child January 19, 1875. The reputed father of the child was at the time a hopeful of fourteen years of age. The child is still alive, but not very strong or bright, though the promising parents are doing as well as could be expected.—*Detroit Medical Journal.*

